# Terminologies matter - the case of ICNP and SNOMED-CT

**DOI:** 10.1093/eurpub/ckac129.364

**Published:** 2022-10-25

**Authors:** A Staines, P Hussey, S Das

**Affiliations:** Centre for eIntegrated Care, Dublin City University, Dublin, Ireland

## Abstract

Terminologies can seem very abstract to end-users. While most health professionals will be familiar with some terminologies (for example MeSH (Medical Subject Headings), the controlled vocabulary thesaurus used for indexing articles for PubMed or ICD-10, WHO's terminology for disease coding), fewer will be aware of the depth and range of terminologies used in healthcare, nor of the central importance of multilingual standard terminologies in health care interoperability. Following a brief introduction to the use of terminologies, the integration of the International Classification for Nursing Practice (ICNP) the Systematized Nomenclature of Medicine - Clinical Terms (SNOMED CT) will be presented, as an example of the use of terminologies, and their ongoing curation, maintenance and development.

